# 

**Published:** 1909-02-15

**Authors:** 


### ANNOUNCEMENTS

NEW JERSEY STATE DENTAL SOCIETY.—This society holds its next annual meeting July 14th to 16th, at Asbury Park. As usual its committee on program is already at work providing a feast of the best things.

ILLINOIS STATE DENTAL SOCIETY.—The forty-fifth annual meeting of the Illinois State Dental Society will be held at Danville, May 11th, 12th, 13th and 14th, 1909.

R. J. HOOD, *Secretary,*
Sparta, Illinois.

ST. LOUIS, MO. SOCIETY OF DENTAL SCIENCE.—This Society held its annual banquet January 19th, at the Jefferson Hotel. An address, by Rev. J. P. Frieden, on True and False Education, and another by Prof. C. M.

Woodward on The Relation of Manuel Training to the Art of Dentistry were important features of the occasion.

DENTAL PEDESTRIAN.—Dr. Alfred Owre, Dean of the University of Minnesota Dental School, walked the 429 miles from Chicago to Minneapolis in 19 days, or 156 hours, exclusive of rests. His average walking time was two and three fourths miles per hour, or about twenty-five miles each day. The Doctor carried his own food in a knapsack, from which he ate four meals each day, saw eighteen sunrises and sets, and slept eighteen nights in hotels, and rested ten minutes out of each hour when walking. He says the great gain he got out of making the trip alone was that of intensifying his own personality, his mind had free play, and nobody or thing worried him during the entire trip..

OFFICERS OF THE ST. LOUIS SOCIETY OF DENTAL SCIENCE.—At the December meeting, the following officers were elected: Dr. W. E. Brown, President; Dr. Clarence O. Simpson, Vice-President; Dr. G. E. Hourn, Secretary; Dr. C. S. Dunham, Treasurer; Dr. A. H. Winklemeyer, Curator. Executive Committee: Dr. E. E. Haverstick, Dr. G. H. Westhoff, Dr. E. J. Lenzen, Dr. Burton Lee Thorpe, Dr. A. H. Winkelmeyer. Advisory Council: Dr. G. A. Bowman, Dr. A. H. Fuller, Dr. D. O. M. LeCron, Dr. Richard Summa, Dr. W. L. Whipple, Dr. H. F. Cassel, Dr. E. P. Dameron.

CORRECTION.—In the November 1908 issue of the REGISTER, at pages 569 and 570, is printed as part of the discussion a quotation from a paper by Dr. E. C. Kirk, which was printed in the *Dental Cosmos* for August, 1908. This quotation was misplaced by the printer in making up the page forms, and should have appeared as a part of Dr. Ward's discussion near the top of page 565.

Dr. Charles R. Butler to be Banqueted.—The Cleveland Dentists will tender Dr. Butler a congratulatory banquet on March 11th, in honor of his long and faithful service in endeavoring to uphold the highest ideals of professional ethics and practice in that city. It ought to be an occasion of great good fellowship and of honoring a most worthy man, who has practiced dentistry more than fifty years. The banquet will be held at the Hollenden Hotel, at 7 o'clock in the evening of March 11th, and all friends of Dr. Butler and the profession in general are cordially invited to participate.

Interstate Dental Fraternity.—The Board of Governors of the Interstate Dental Fraternity will convene for the annual business meeting of the Order at Old Point Comfort, August 1st, 1909. The annual banquet will occur during the week and due notice thereof will be sent to the members as soon as arrangements can be made and the exact date fixed. It is hoped that the Fraternity will meet in large numbers on this occasion.

Dr. R. M. Sanger, *National Secretary,*
East Orange, N. J.

First District Dental Society's Annual Clinic.—The annual clinic of this society was held February 13th, 1909, at the Detroit Dental College. The address for the occasion was by Dr. F. B. Noyes, of Chicago. The clinics were numerous and of a high order of skill and attainment.

---

THE MARQUETTE, MICHIGAN, DENTAL SOCIETY.—The dentists of the district about Marquette, Mich., on January 2nd, met in Negaunee and organized a dental society, which they hope will include all the dentists of that vicinity and become very soon one of the component district societies of the Michigan State Dental Society.

---

SUPERNUMERARY TEETH.—Dr. G. V. Black exhibited as the recent meeting of the Institute of Dental Pedagogics a large number of specimens of supernumerary teeth and other unusual specimens of teeth from the Museum of the Northwestern University Dental School. They were exceedingly well mounted and attracted great attention.

---

THE RANSOM AND RANDOLPH CO.—This Company has taken over the business of T. O. Tracy & Co., of Grand Rapids Mich. Mr. Tracy will hereafter be a member of the firm and have charge of the retail department at Toledo, and serve the Company as its Vice President. Mr. Ray E. Munn, for many years a well-known salesman of this house also enters the firm and takes charge of the branch house at Grand Rapids, while Mr. Fred Bouse will have charge of the Ann Arbor branch. The new Company has taken on a lot of new enterprises and expects to make its mission well known throughout the middle west. The *Dental Summary*, published by this house, has also undergone a great transformation, and it is the intention of the firm to make it the best journal published. It certainly starts the new year promisingly. We wish the new house and its undertakings abundant success.

CHICAGO ODONTOGRAPHIC SOCIETY.—On January 12th and 13th, this society celebrated its *one thousandth membership jubilee* in a clinic of two days. The program was for a manufacturers exhibit and symposium on operative dentistry for the first day and a clinic and symposium on prosthetic dentistry and oral surgery for the second day. There was a very large attendance and a most successful gathering of clinicians. As in all such large gatherings the value of the clinics were not all available to all who attended, yet every one got enough to compensate him for attending and the society is to be congratulated on its very large membership and ability to get together so many excellent clinics and so enthusiastic an attendance.

